# Establishment and characterization of urothelial carcinoma cell lines with and without *BRAF* mutation (V595E) in dogs

**DOI:** 10.1007/s11626-022-00736-0

**Published:** 2022-12-07

**Authors:** Hirofumi Yamasaki, Yosuke Uematsu, Kumiko Okano, Mika Ichikawa, Meina Tei, Miyuki Hirabayashi, Kazuyuki Uchida, Kenichiro Ono, Hidehiro Hirao

**Affiliations:** 1 Japan Animal Referral Medical Center, 2-5-8 Kuji, Takatsu-Ku, Kawasaki-Shi, Kanagawa, 213-0032 Japan; 2 Canine-Lab, 3-5-2 Ueno, Taito-Ku, Tokyo, 110-0005 Japan; 3 Department of Veterinary Pathology, Faculty of Agricultural and Life Sciences, The University of Tokyo, 1-1-1 Yayoi, Bunkyo-Ku, Tokyo, 113-8657 Japan

**Keywords:** BRAF gene nutation (V595E), Cell line, Dog, Phosphorylated-BRAF and phophorylated-ERK1/2, Urothelial carcinoma

## Abstract

Each 5 urothelial carcinoma (UC) cell lines with and without the v-Raf murine sarcoma virus oncogene homolog B (BRAF) gene mutation (V595E) were established and examined V595E-related tumorigenic characteristics in dogs. No typical morphological features were observed in cloned cells with and without V595E. The cell proliferation of both cloned cells showed logarithmic growth curve and those doubling time were 24.9 ± 4.1 h in V595E ( +) and 29.3 ± 11.3 h in V595E ( −). On the growth curve of xenotransplanted tumor in severe combined immunodeficiency mice, 3 out of 5 V595E ( +) and 2 out of 5 V595E ( −) cloned cells revealed gradually and remarkably increasing curve, indicating clearly tumorigenicity. The xenotransplanted tumors with V595E ( +) showed typical features of UC, such as solid proliferation of pleomorphic tumor cells, formation of papillary structure, and glandular structure. Additionally, various vascular formation was observed, probably indicating an advanced growth phase of UC. In mitogen-activated protein kinase (MAPK) signaling pathway, cytoplasmic phosphorylated-BRAF (pBRAF) and cytoplasmic and nuclear phosphorylated-ERK1/2 (pERK1/2) were detected in all 4 tumors with V595E ( +), whereas only cytoplasmic and nuclear pERK1/2 was detected in tumors with V595E ( −). Since V595E can directly activate MAPK signaling pathway, coincidence of V595E with pBRAF (phosphor Thr598/Ser601) indicates acquired resistance to BRAF inhibitors. These established UC cell lines, especially V595E ( +) cell lines, are useful tool for understanding pathophysiological states and controlling therapeutic manners of UC in dogs.

## Introduction

Urothelial carcinoma (UC), well known as transitional cell carcinoma, is the most common tumor in the urinary tract, especially urinary bladder in dogs. The tumor, originating from urothelial epithelium cells, shows highly invasive behavior into the lamina propria and distant metastasis (Reed *et al*. 2012; Knapp *et al*. 2014; Fulkerson and Knapp 2015). Indeed, metastatic lesions were observed in 20% of dogs with UC at the time of diagnosis and in up to 50% of dogs at the death (Mutsaers *et al*. 2003; Allstadt *et al*. 2015; Mochizuki *et al*. 2015). Although various predispositions, such as age, breed, sex, and obesity were reported, the most important prognostic factor is metastatic dynamics of the tumor cells (Norris *et al*. 1992; Knapp *et al*. 2000; Griffin *et al*. 2018). As median survival time (MST) was reported by 3.5–4.0 months after the surgical treatment (Mutsaers *et al*. 2003; Griffin *et al*. 2018), similar MST (2.5–4.5 mo) was reported with the carboplatin treatment. The UC commonly shows poor response to chemotherapy and poor prognosis (Allstadt *et al*. 2015; Fulkerson and Knapp 2015).

Recently, the v-Raf murine sarcoma virus oncogene homolog B (BRAF) gene mutation in exon 15 (V595E), corresponding to human V600E, was identified and detected in various tumors in dogs, especially in UC with high prevalence rates of 67 to 87% (Decker *et al*. 2015; Mochizuki *et al*. 2015). It is well known that BRAF is one of the most important serine/threonine kinase and activates mitogen-activated protein kinase (MAPK) signaling pathway. The V595E can directly phosphorylate and activate its downstream MAPK kinase (MEK), followed by phosphorylated and activated extracellular single-regulated kinase (ERK1/2), indicating oncogenicity and tumorigenicity of UC as the driver mutation (Decker *et al*. 2015; Mochizuki *et al*. 2015; Mochizuki and Breen 2015). Human colorectal tumors with *BRAF* mutation (V600E) show histologically high grade, poor survival time, and increasing microsatellite instability and/or frequent DNA methylation related with tumorigenicity (Nagasaka *et al*. 2004; Li *et al*. 2006; Jung *et al*. 2021). In contrast, our report demonstrated that V595E coincided with phosphorylated BRAF (pBRAF), indicating key of the resistance to BRAF inhibitors, in all 13 formalin-fixed tissue samples of UC with V595E ( +) (Yamasaki *et al*. 2022).

On the other hand, tumor cell lines are one of the most valuable tools for understanding morphological differentiation, mechanism of cell growth, biological behavior, tumorigenicity of neoplastic cells, and responses with anti-tumor drugs (Choi *et al*. 2014; Kito *et al*. 2018; Packeiser *et al*. 2020). However, there are few reports on cell lines established from original UC with V595E ( +) in dogs (Jung *et al*. 2021). In the present study, each 5 cell lines from UC with and without V595E were established and examined on morphological features, cell proliferation, tumorigenicity, and expression of phosphorylated-BRAF and phosphorylated-ERK1/2 in xenotransplanted tumors.

## Materials and methods

### Clinical characteristics of original tumor samples for establishment of UC cell lines

**Figure 1.**
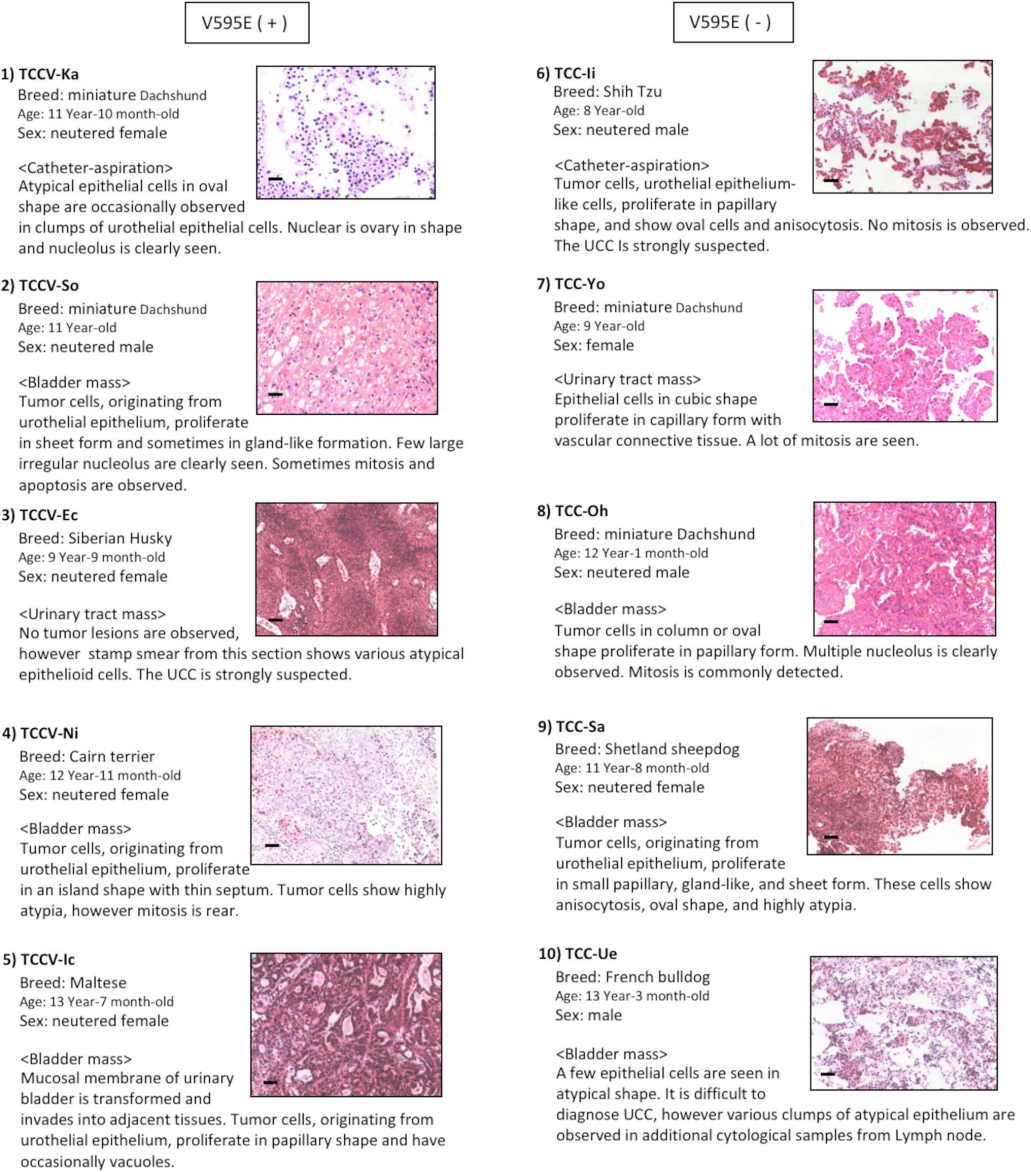
Clinical characteristics of tissue samples for establishment of cell lines from urothelial carcinoma (UC) with and without *BRAF* mutation (V595E) in dogs. Four breeds, consisted of 2 miniature Dachshund, Siberian Husky, Cairn terrier, and Maltese, are observed in V595E ( +) cases, whereas 4 breeds, consisted of 2 miniature Dachshund, Shih Tzu, Shetland sheepdog, and French bulldog, are observed in V595E ( −). The age ranged from 9 years and 9 months old to 13 years and 7 months old in V595E ( +) and from 8 years old to 13 years and  3 months old in V595E ( −). Gender of each 5 cases of V595E ( +) and V595E ( −) is 4 neutered female and 1 neutered male dog, and 1 neutered female, 1 intact female, 2 neutered male, and 1 intact male dog, respectively. There are no remarkable differences on histological or cytological features between V595E ( +) and V595E ( −) samples. Both catheter-aspirations of V595E ( +) and V595E ( −) reveal atypical epithelial cells in oval shape and proliferation of tumor cells in papillary shape with anisocytosis (TCCV-Ka, TCC-Ii). Proliferation of tumor cells in sheet formation (TCCV-So, TCC-Sa), various atypical epithelioid cells (TCCV-Ec, TCC-Ue), in an island form (TCCV-Ni), and in papillary form (TCCV-Ic, TCC-Yo, TCC-Oh) are observed

A total of 10 tissue samples of UC, including 2 catheter-aspiration specimens, were used. All tissue samples confirmed diagnosis or strongly suspected, based on their histopathological or cytological findings, by 2 board-certified veterinary pathologists at the Japanese College of Veterinary Pathologists. The UC tissue samples were subdivided into each 5 cases of *BRAF* mutation with and without V595E, abbreviated as V595E ( +) and V595E ( −), respectively. The V595E ( −) means wild-type gene of BRAF. Clinical characteristics of these samples are represented on breed, age, sex, and histopathological or cytological findings in Fig. 1. Most of UC tissue samples revealed some typical histopathological or cytological features of UC, such as proliferation of neoplastic cells in papillary shape, in oval cells with anisocytosis, and in gland-like formation. There were no remarkable differences of clinical characteristics between V595E ( +) and V595E ( −) with an exception of the sex. In V595E ( +), 4 out of 5 cases were neutered female dogs, and the remaining was neutered male, whereas one intact male, two neutered male, one intact female, and one neutered female were observed in V595E ( −).

### Analysis of BRAF mutation (V595E)

**Figure 2.**
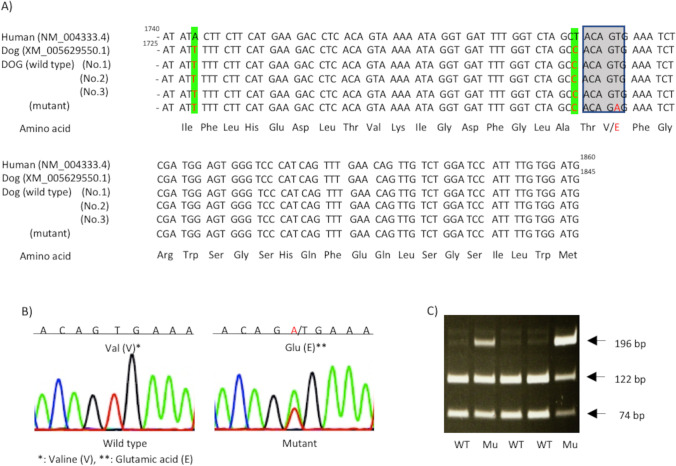
Genomic DNA sequence, PCR-sequencing chromatogram, and PCR–RFLP electropherogram of *BRAF* exon 15 gene with mutation (V595E) and wild type. (*A*) DNA sequence of wild type (WT) of BRAF gene in human (NM_004333.4) (*upper 1st line*) and in dog (XM_005629550.1) (*2nd line*). Using primer pairs mentioned in the “Materials and methods” section, the sequences are highly conserved between human and dogs (Nos. 1–3), although 2 silent point mutations are observed (*green band*). DNA fragment of 196 base pair (bp), corresponding to human *BRAF* exon 15 gene, is amplified and sequenced. Squared 5 genomic bases, in which latter 3 bases consist of codon 595 and include mutant sequence (red character; 6th line), are the digestion site of BtsIMutI restriction enzyme. (*B*) Chromatogram of the wild type (left) and mutant (right) sequence of *BRAF* in dogs. When V595E with substitution of c.1784 T > A, corresponding to human V600E with substitution of c.1799 T > A, is developed, the restriction enzyme site, sequencing CAGTG, disappeared. (*C*) V595E is clearly detected on PCR–RFLP electropherogram. The amplicons are digested to 2 fragments of 122 and 74 bp in wild-type sequence, whereas amplicon is not digested in mutant sequence, demonstrating 3 bands of 196-, 122-, and 74-bp fragment

The V595E mutation was analyzed by polymerase chain reaction-restriction fragment length polymorphism (PCR–RFLP) (Decker *et al*. 2015; Mochizuki *et al*. 2015; Jung *et al*. 2021; Yamasaki *et al*. 2022). Using primer pairs, reverse primer 5′-TGG CCT CAA TTC TTA CCA TCC AC-3′, designed by Decker *et al*. (2015), and forward primer 5′-GTA ATG CTT GCT TTG CTA GGA 3′, originally designed from genomic DNA, based on GenBank (accession No. NC_006598.2), 196 base pair (bp) DNA fragment, corresponding to human BRAF gene exon 15, was amplified (Yamasaki *et al*. 2022). When V595E with substitution of nucleotide (c.1784 T > A), corresponding to human V600E with substitution of c.1799 T > A, was developed, the location of restriction enzyme site with BtsIMutl (New England Biolabs Japan Inc., Tokyo, Japan), sequencing CAGTG, disappeared (Fig. 2*A*, *B*). As PCR amplicons of wild-type BRAF gene were digested by BtsIMutI, V595E was clearly detected by RFLP analysis. Briefly, the amplicon of wild-type BRAF gene is digested into 2 DNA fragments of 122 and 74 bp, whereas BRAF mutant gene (V595E) is not digested, indicating 3 fragments (196, 122, and 74 bp) (Fig. 2*C*).

### Establishment of cell lines (cell culture and cloning)

The tissue or catheter aspiration samples were cut, minced, dissolved, and seeded in 90-mm tissue culture dish (Nunclon, Thermo Fisher Scientific, Waltham, MA). The tumor cells were maintained in a humidified atmosphere 5% CO2 at 37 °C in Dulbecco’s modified Eagle’s medium (Gibco, Thermo Fisher Scientific) supplemented with 20% fetal bovine serum (Gibco, Thermo Fisher Scientific) and 1% penicillin–streptomycin (Sigma, Sigma-Aldrich, Saint Louis, MO). The cells were passaged when they reached confluence. After 50–60 passage cultivation, cloned cells were obtained by the limiting dilution culture

### Cell proliferation assay (doubling time)

Cell proliferation assay was performed for calculating of the doubling time. The cloned tumor cells from UC with V595E ( +) and V595 ( −) were cultured in 12-well culture plate (Falcon, Corning Incorporated Life Sciences, Tewksbury, MA) at 1.5 × 10^5^ cells/well to obtain the growth curve. The cells were counted on hemocytometer of every day in triplicate wells from day 1 to day 5.

### Xenotransplantation of the cloned UC cells into SCID mice

Each 5 cloned tumor cells with V595E ( +) and V595E ( −) were subcutaneously inoculated with 0.22 − 8.0 × 10^7^ cells in 200 μl of physiological saline (TERUMO Inc, Tokyo, Japan) into the lower flunk of each 3 female 5-wk-old severe combined immunodeficiency (SCID) mice (total 30 mice) (FOX CHASE SCID CB-17/lcr-scid/scidJcl; CLEA Japan Inc., Tokyo, Japan). The inoculated mice were housed in a cage (3 mice/cage) with free access to water and a standard purified rodent growth diet (AIN-93G, Oriental Yeast, Tokyo, Japan). When the mass was palpable at the inoculation site, designed as day 0 after the inoculation in this experiment, the tumor volume (length × width × height) was measured every day, using a digital vernier caliper. At the 28th–35th day after the inoculation, the mice were euthanized with isoflurane and the tumor was resected for histopathological and immunohistochemical examinations.

All animal experiments were approved by the Committee of Animal Experiments, Graduate School of Agricultural and Life Sciences, The University of Tokyo (approve Number P17-017).

### Histopathological and immunohistochemical analysis for xenotransplanted tumor

For histopathological analysis, total 9 xenotransplanted tumor samples, consisted of 4 with V595E ( +) and 5 with V595E ( −) tumor tissues, were fixed in 10% neutral buffered formalin, routinely paraffin processed, sectioned at 4 μm, and stained with hematoxylin and eosin (H&E). For immunohistochemical analysis of phosphorylated-BRAF (pBRAF) and phosphorylated-ERK1/2 (pERK1/2), 4-μm tissue sections of xenotransplanted tumor were used. Briefly, antigen retrieval was performed by autoclaving tissue sections in citrate buffer (pH 6.0) at 121 °C for 10 min. Endogenous peroxidase activity in the tissue sections was inactivated with 3% hydrogen peroxide in methanol at room temperature for 5 min. To block nonspecific reactions, the sections were incubated with 8% skimmed milk in Tris-buffered saline (TBS) at 37 °C for 30 min. The sections were then incubated at 4 °C overnight with each primary antibody: rabbit polyclonal anti-BRAF (phosphor Thr598/Ser601) (Cat. GTX85596, 1:100, Gene Tex Inc., Irvine, CA) and rabbit monoclonal anti-phospho-p44/42 MAPK (Erk 1/2) (Thr 202/Tyr204) (20G11) (Cat. #4376S, 1:400, CST Japan, Tokyo, Japan). The primary antibodies were replaced with TBS to produce a negative control. After 3 washings with TBS, the sections were treated with Dako EnVision + System-horseradish peroxidase-labeled polymer anti-rabbit secondary antibodies (Agilent Technologies Japan Ltd, Tokyo, Japan) at 37 °C for 40 min. The chromogen consisted of 0.05% 3–3′-diaminobenzidine and 0.03% hydrogen peroxide in Tris–HCl buffer. The sections were counterstained with Mayer’s hematoxylin.

## Results

### Establishment of cell lines (cell culture and cloning)

**Figure 3.**
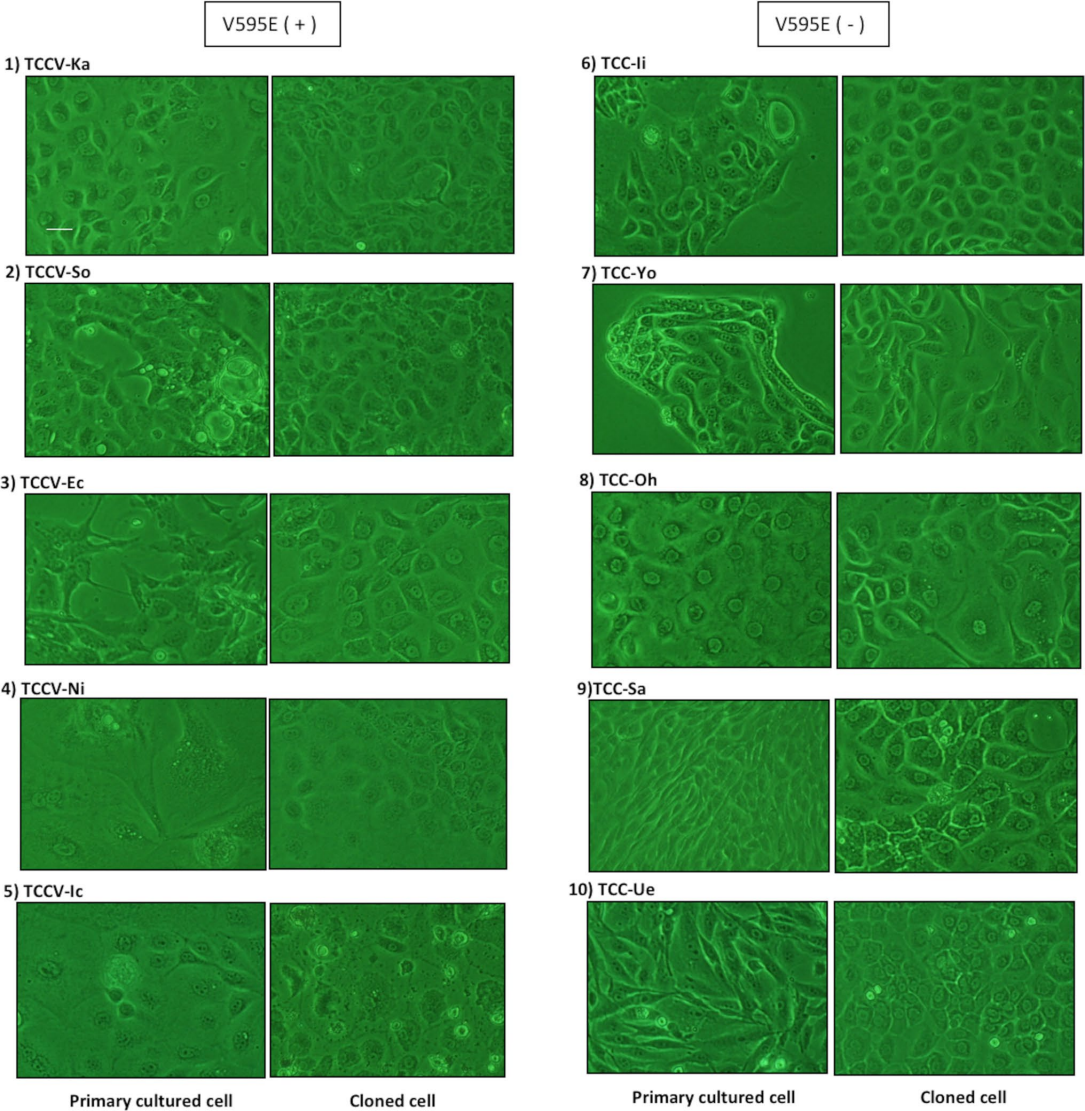
Primary cultured cells and cloned cells from UC with V595E ( +) and V595E ( −). Inverted microscopic images of primary cultured cells and cloned cells from UC with V595E ( +) are represented in the *1st* and the *2nd columns*, respectively, and those with V595E ( −) are represented in the *3rd* and the *4th columns*, respectively. The primary cultured cells with V595E ( +) are grown in adherent and monolayer cells with spindle and round cell shape (TCCV-Ka), round and large polygonal cell shape (TCCV-So, TCCV-Ec), large polygonal cell shape with polynuclear (TCCV-Ni), and round and large polygonal cell shape (TCCV-Ic), whereas the cells with V595E ( −) are grown in island formation (TCC-Ii, TCC-Yo), round to oval cell shape (TCC-Oh), and spindle cell shape (TCC-Sa, TCC-Ue). The cloned cells with V595E ( +) are grown round to oval and large polygonal cell shape (TCCV-Ka), round to oval cell shape (TCCV-So), round cell shape (TCCV-Ec), round and squamous cell shape (TCCV-Ni), and squamous and large polygonal cell shape (TCCV-Ic), whereas the cells with V595E ( −) are grown in round to oval cell shape (TCC-Ii), spindle-like and large polygonal cell shape (TCC-Yo), squamous and large polygonal cell shape (TCC-Oh), and squamous cell shape (TCC-Sa, TCC-Ue). There are no remarkable morphological differences in cloned cells between V595E ( +) and V595E ( −). *Bar* = 20 μm

Figure 3 shows inverted microscopic images of both primary cultured cells (passage number: 3–5) and cloned cells (cloned by the limiting dilution method) of UC with V595E ( +) (1st and 2nd column, respectively) and with V595E ( −) (3rd and 4th columns, respectively). The primary cultured cells from UC with V595E ( +) consisted of spindle cells (fibroblast-like cells), oval to round cells (epithelial-like cells), and sometimes polygonal and giant cells. In addition, those from UC with V595E ( −) revealed island formation (2 cases), and consisted of spindle cells and oval to round cells. In cloned cells, there were no remarkable morphological differences between V595E ( +) and V595E ( −) cells, such as round to oval cells (epithelial-like cells) and sometimes with large polygonal cells. Morphological features (cell shape) are summarized in Table 1.

**Table 1:** Morphological features in primary cells and cloned cells

	Primary cells	Cell shape	Cloned cells	Cell shape
	1)TCCV-Ka	Spindle and round	1)TCCV-Ka	Round and large polygonal
	2) TCCV-So	Large polygonal	2) TCCV-So	Round to oval
V595E ( +)	3) TCCV-Ec	Large polygonal	3) TCCV-Ec	Round
	4) TCCV-Ni	Large polygonal with polynuclear	4) TCCV-Ni	Round and squamous
	5) TCCV-Ic	Round and large polygonal	5) TCCV-Ic	Squamous and large polygonal
	6)TCC-Ii	Island formation	6)TCC-Ii	Round and oval
	7) TCC-Yo	Island formation	7) TCC-Yo	Spindle-like and large polygonal
V595E (-)	8) TCC-Oh	Round and oval	8) TCC-Oh	Squamous and large polygonal
	9) TCC-Sa	Spindler	9) TCC-Sa	Squamous
	10) TCC-Ue	Spindle	10) TCC-Ue	Squamous

### Cell proliferation assay (doubling time)

**Figure 4.**
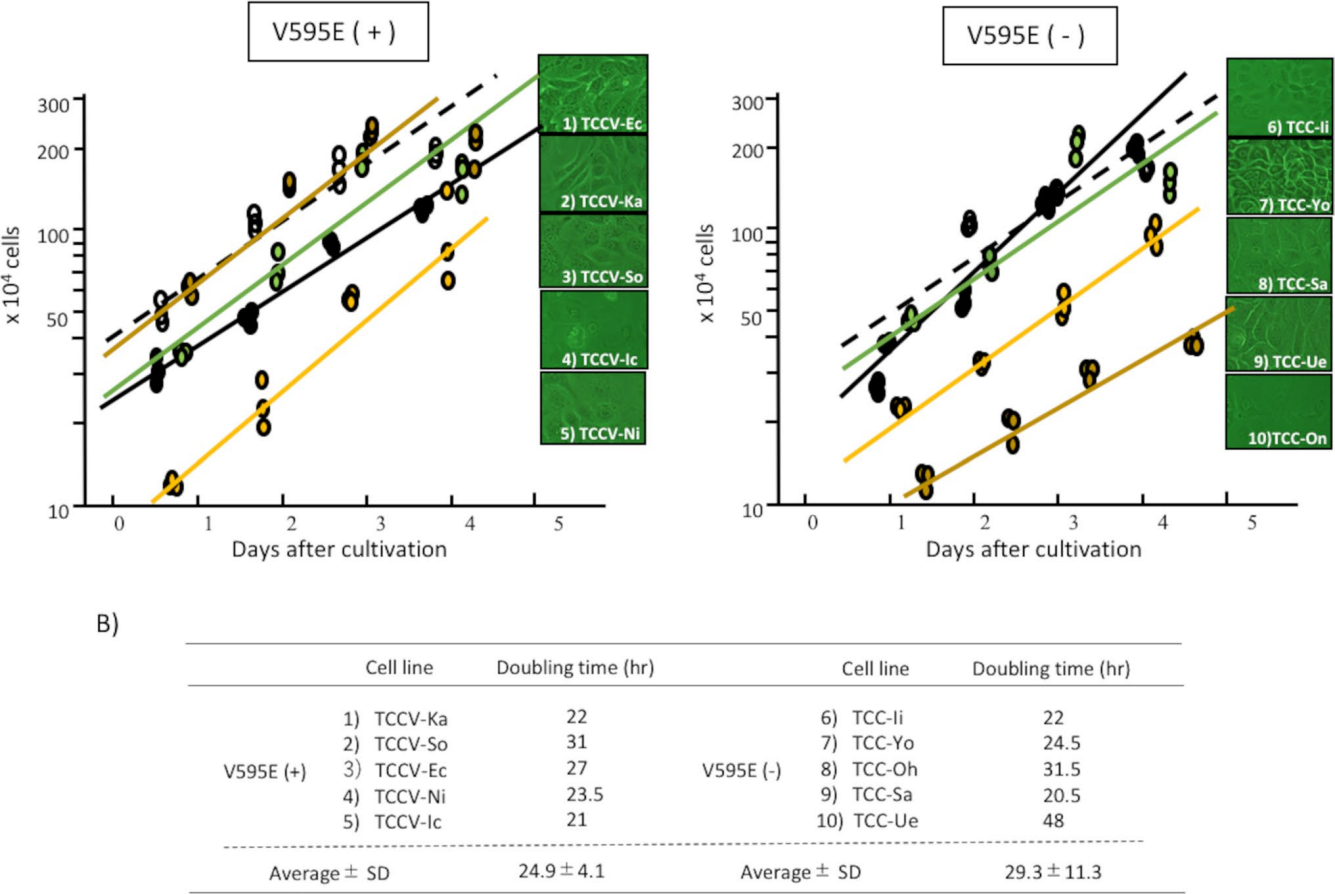
Cell proliferation assay (doubling time) of the cloned cells from UC with V595E ( +) and V595E ( −). Each 5 cloned cells from both UC with V595E ( +) and V595E ( −) show similar logarithmic cell growth (*A*: *left* and *right graph*, respectively). Calculating those doubling time are summarized in the table (*B*)

Both cloned cells from UC with V595E ( +) and V595E ( −) showed similar logarithmic growth curve (Fig. 4*A*; left and right graph, respectively). Those calculating doubling time are summarized in Fig. 4 *B*. The doubling time of the cloned cells with V595E ( +) and V595E ( −) were 24.9 ± 4.1 h and 29.3 ± 11.3 h (average ± SD), respectively.

### Xenotransplantation of the cloned UC cells into severe combined immunodeficiency mice

**Figure 5.**
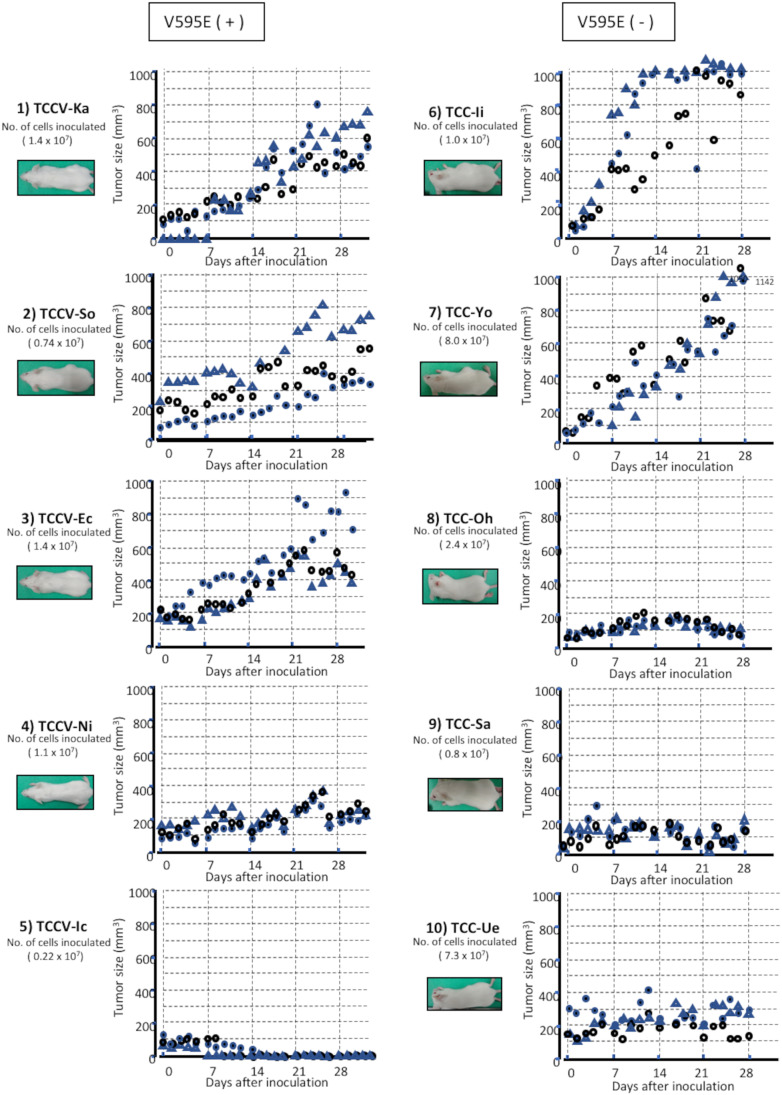
Tumor growth curve of cloned UC cells with V595E ( +) or V595E ( −) in subcutaneously xenotransplanted into SCID mice. There are 3 types of tumor growth curve, such as gradually and remarkably increasing tumor size, approximately maintaining tumor size at day 0, and gradually decreasing in tumor size. The SCID mice, inoculated with 3 out of 5 cloned cells with V595E ( +), show gradually and remarkably increasing tumor size (TCCV-Ka, TCCV-So, and TCCV-Ec). One of the remaining 2 SCID mice, TCCV-Ni reveals approximately maintaining tumor size at day 0 and another TCCV-Ic reveals decreasing in tumor size by day 14 (left graph), whereas 2 out of 5 SCID mice, inoculated with cloned cells with V595E ( −), show gradually and remarkably increasing tumor size (TCC-Ii and TCC-Yo). The remaining 3 SCID mice (TCC-Oh, TCC-Sa, and TCC-Ue) reveal maintaining tumor size (right graph)

There were 3 types of growth curve, such as gradually and remarkably increasing, approximately maintaining tumor size at day 0, and gradually decreasing, in xenotransplanted tumors. The SCID mice, inoculated with 3 cloned cells with V595E ( +), showed gradually and remarkably increasing curve. One of the remaining 2 SCID mice revealed approximately maintaining curve and the other mice revealed gradually decreasing curve by day 14 (Fig. 5, left graph), whereas 2 SCID mice, inoculated with cloned cells with V595E ( −), showed gradually and remarkably increasing curve. The remaining 3 SCID mice revealed approximately maintaining curve (Fig. 5, right graph).

### Histological and immunohistochemical features of xenotransplanted tumor, inoculated cloned cells with V595E ( +) or V595E ( −)

**Figure 6.**
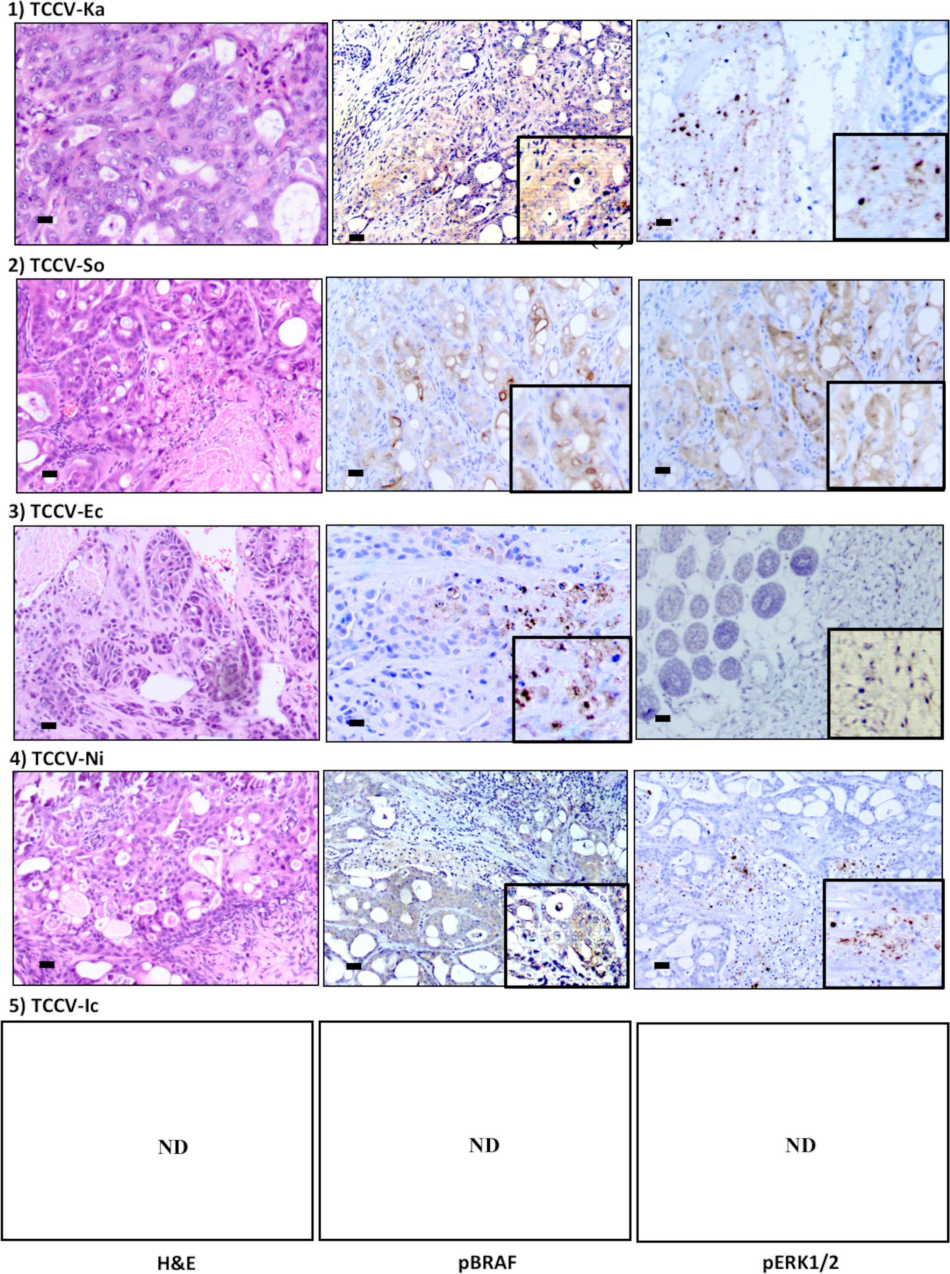
Histopathological futures and expression of phosphorylated-BRAF (pBRAF) and phosphorylated-ERK1/2 (pERK1/2) in xenotransplanted tumors with V595E ( +) cloned cells. The 4 cases of xenotransplanted tumors, with the exception of decreasing case (TCCV-Ic), reveal some typical histopathological features of UC, such as the formation of neoplastic foci (TCCV-Ka and TCCV-So), formation of glandular structure with tumor cells (TCCV-Ec), and solid proliferation of pleomorphic tumor cells (TCCV-Ni). Additionally, various vacuoles are observed in 3 cases (TCCV-Ka, TCCV-So, and TCCV-Ni) (left column; H&E staining, *Bar* = 50 μm). On the results of immunohistochemistry, cytoplasmic immunoreactivity against pBRAF is detected in all 4 cases examined. Although nuclear immunoreactivity is detected in 2 cases (TCCV-Ka and TCCV-Ec), these reactivities are non-specific, due to pBRAF being limited in cytoplasm (center column, *bar* = 50 μm). Additionally, cytoplasmic immunoreactivities against pERK1/2 are detected in all 4 cases with positive reactions of nuclear immunoreactivity (right column, *bar* = 50 μm)

With an exception of the decreasing case, 4 xenotransplanted tumors, inoculated cloned cells with V595E ( +), showed some typical histopathological features of UC, such as formation of neoplastic foci, solid proliferation of pleomorphic tumor cells, formation of papillary structure toward the lumen, and glandular structure with tumor cells. In addition, various vascular formation was observed in 3 cases (Fig. 6, left column). Cytoplasmic immunoreactivity against pBRAF (phosphor Thr598/Ser601) was detected in all 4 cases examined. Although nuclear immunoreactivity was detected in 2 cases, these reactivities were non-specific, due to pBRAF being limited in cytoplasm (center column). Cytoplasmic immunoreactivities against pERK1/2 (Thr 202/Tyr204), associated with nuclear immunoreactivity, were detected in all 4 cases, indicating activation of MAPK signaling pathway (right column).

**Figure 7.**
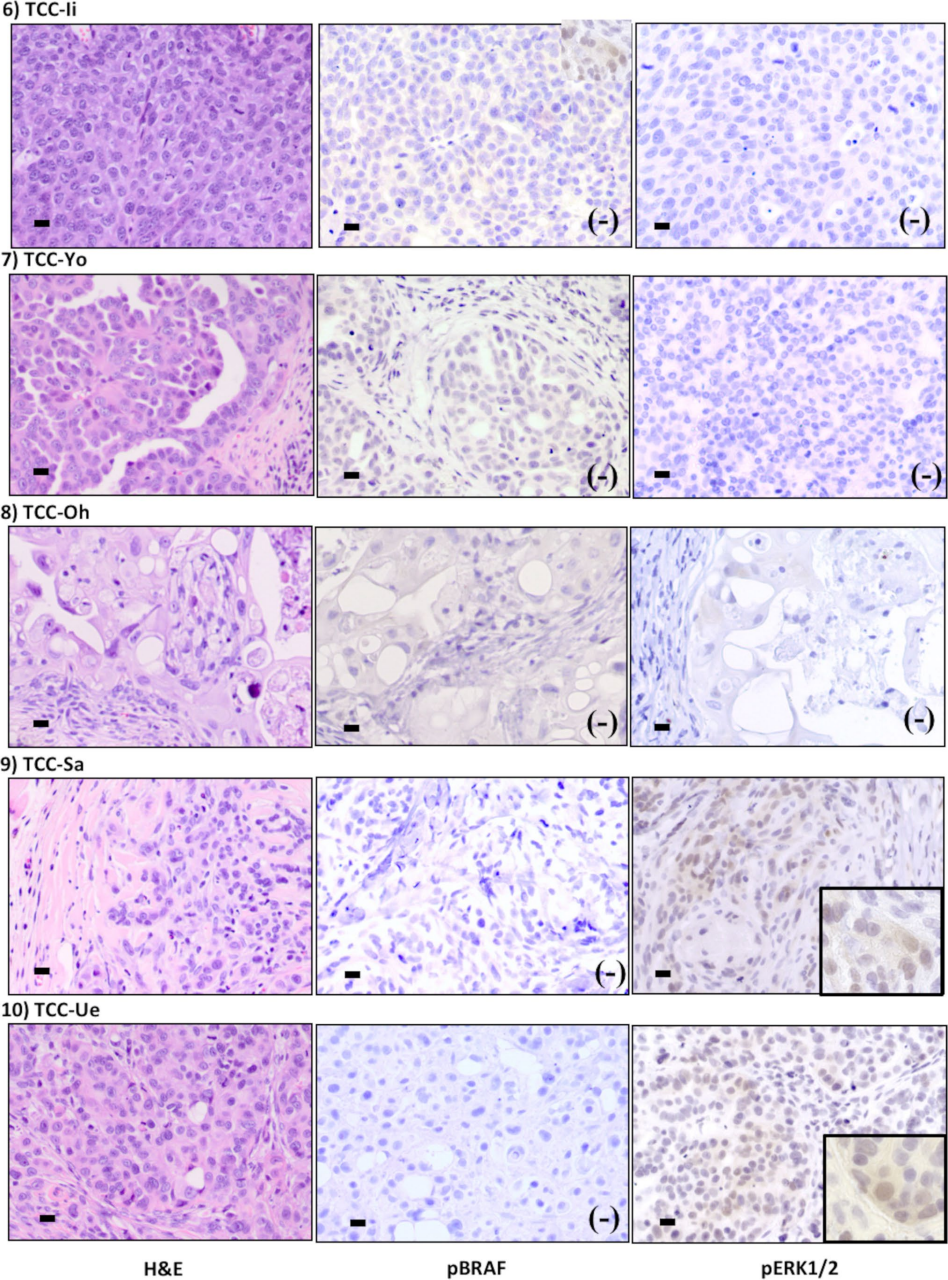
Histopathological futures and expression of phosphorylated-BRAF (pBRAF) and phophorylated-ERK1/2 (pERK1/2) in xenotransplanted tumors with V595E ( −) cloned cells. All 5 tumors reveal typical histopathological features of UC, such as proliferation of the pleomorphic tumor cells in sheet formation (TCC-Ii) and sometimes in gland-like formation (TCC-Ue), papillary structure toward the lumen (TCC-Yo), formation in island shape with thin septum (TCC-Oh), and formation in glandular structure (TCC-Sa) (*left column*, H&E *staining bar* = 50 μm). None of positive cytoplasmic immunoreactivity against pBRAF is detected, whereas cytoplasmic immunoreactivity against pERK1/2 is detected in 2 cases (TCC-Sa, TCC-Ue) (center and right column, respectively, *bar* = 50 μm)

Figure 7 shows xenotransplanted tumors, inoculated with cloned cells of V595E ( −). All 5 tumors revealed typical histopathological features of UC, such as the proliferation of the pleomorphic tumor cells in sheet formation, sometimes in gland-like formation, formation in papillary structure toward the lumen, formation in island shape with thin septum, and formation in glandular structure (left column). None of positive cytoplasmic immunoreactivity against pBRAF was detected, whereas cytoplasmic immunoreactivity against pERK1/2 was detected in 2 cases (center and right column, respectively).

## Discussion

It is widely accepted that V595E is the most important oncogenic mutations of BRAF gene in dogs, corresponding to V600E in human tumors. The V595E is the driver mutation of canine UC with extremely high prevalence rates and activates MAPK signaling pathway, indicating tumorigenicity of UC (Decker *et al*. 2015; Mochizuki *et al*. 2015). The PCR–RFLP analysis, as shown in this study, is available tool for detection of V595E, providing genomic DNA sequence and PCR sequencing chromatogram of V595E. The V595E is closely associated with morphology, cell growth, tumorigenesis, metastatic mechanism, and potential of chemotherapy of UC, like as reported in the tumor with V600E (Garnett and Marais 2004; Li *et al*. 2006; Jung *et al*. 2021). Tumor cell lines are valuable tool for understanding cell growth, biological behavior, tumorigenicity, and responses with anti-tumor drugs (Choi *et al*. 2014; Kito *et al*. 2018; Packeiser *et al*. 2020).

Although 15 cell lines of canine UC are listed in bladder cancer (Dhawan *et al*. 2009; Cekanova *et al*. 2013; Decker *et al*. 2015; Zuiverloon *et al*. 2018), only 2 cell lines are established from original tumors with V595E ( +) and examined their characteristics (Jung *et al*. 2021). In this study, each 5 newly cell lines from UC with and without V595E were established. Both primary cultured cells with and without V595E revealed adherent monolayer cells and sometimes island formation. They revealed heterogeneous cell in shape, such as fibroblast-like cells, epithelial-like cells, round cell, and sometimes giant cells, probably because originating parent tumor consisted of different growth phase of cells (Palyi *et al*. 1977; Jung *et al*. 2021). There were no typical morphological features related with V595E of cloned cells.

In cell proliferation, both cloned cells with and without V595E showed similar logarithmic growth curve and doubling time, calculating as 24.9 ± 4.1 h in V595E ( +) and 29.3 ± 11.3 h in V595E ( −). Although slightly shorter doubling time (ranged 17.7–20.0 h) was reported in UC with V595E ( +), these values were overlapped with wide range of doubling time in UC with V595E ( −) (Dhawan *et al*. 2009; Zuiverloon *et al*. 2018; Jung *et al*. 2021). In growth curve of xenotransplanted tumor, subcutaneously inoculated with both cloned cells with and without V595E into SCID mice, 3 out of 5 tumors with V595E ( +) and 2 out of 5 tumors with V595E ( −) revealed gradually and remarkably increasing of tumor size, indicating strong tumorigenic activity. As the tumorgenicity is closely related to invasion and metastasis, poor prognosis commonly observed in UC might be induced by their activity (Norris *et al*. 1992; Knapp *et al*. 2000; Griffin *et al*. 2018). On the other hand, one xenotransplanted tumor with V595E ( +) gradually decreased during the experimental period; like as those in previous reports. Tumor formation required some interaction with fibroblasts, macrophages, and adjacent connective tissues (Dhawan *et al*. 2009; Rathore and Cekanova 2014; Jung *et al*. 2021). Xenotransplanted tumors with V595E ( +) showed some typical histopathological features of UC, such as the formation of neoplastic foci, solid proliferation, and formation of papillary structure and/or glandular structure. Various vascular formation was observed in 3 out of 4 tumors. Whereas all 5 xenotransplanted tumors with V595E ( −) also revealed typical histopathological features of UC, such as sheet formation, sometimes in gland-like formation, papillary structure, and island in shape, these features might be reflected early growing stage of UC (Li *et al*. 2006; Patrick *et al*. 2006; Grassinger *et al*. 2019).

In MAPK signaling pathway, all 4 tumors with V595E ( +) revealed cytoplasmic immunoreactivity against pBRAF (phosphor Thr598/Ser601). Nuclear immunoreactivity against pBRAF was also detected in 2 tumors; however, nuclear reactivities were non-specific, due to pBRAF being fundamentally limited in cytoplasm (Davies *et al*. 2002; Loo *et al*. 2018). In contrast, no cytoplasmic pBRAF immunoreactivity was detected in all tumors with V595E ( −). Phosphorylation sites at Thr598/Ser601 in BRAF are major and essential sites of the activated RAS (rat sarcoma viral oncogene homolog) signaling, consisting of RKT (receptor tyrosine kinase)-RAS-BRAF-MEK-ERK cascade (Zhang and Guan 2000, 2001). It is well known that V595E, like as other *BRAF* mutations, can directly phosphorylate and activate MEK with subsequent activation of ERK1/2, by changing of the conformation in activating loop of BRAF (Liu *et al*. 2019; Maloney *et al*. 2021). In all 4 tumors with V595E ( +), both cytoplasmic and nuclear immunoreactivity against pERK1/2 (phosphor-p44/42) were detected, indicating tumorigenicity of the tumor. As nuclear pERK1/2, translocated from cytoplasm, indicates progressive signaling process in MAPK pathway, UC with V595E ( +) might be growing stage of the tumor (Parikh *et al* 2012; Maik-Rachline *et al* 2019).

Interestingly, UC with V595E ( +) constantly shows pBRAF, as the same in our previous study in formalin fixed 13 UC tissue samples (Yamasaki *et al*. 2022) and in 2 cell lines reported by Jung *et al*. (2021). The BRAF gene mutation (V600E) never coincides with activated RAS and RTK, both of which induces pBRAF, in human tumors (Haling *et al*. 2014; Mochizuki and Breen 2017; Dvorak *et al*. 2019). In contrast, many researchers reported that pBRAF, detected in the melanoma with acquired resistant to BRAF inhibitors, is caused by paradoxically activation of upstream RTK or RAS through ERK1/2 feedback mechanism, suggesting new strategies for UC chemotherapy (Nazarian *et al*. 2010; Little *et al*. 2011; Prahallad *et al*. 2012; Holderfield *et al*. 2013). When activation of MAPK signaling pathway as pERK1/2 without pBRAF was observed in UC with V595E ( −), activation of RTK-RAS-PI3K (phosphatidylinositol-3-kinase)-mTOR (mammalian target of rapamycin) cascade, or GPCR (G protein–coupled receptor)-GNAG (G protein: guanine nucleotide binding protein)-MEK-ERK1/2 cascade might be related with tumorigenicity of UC (Porta *et al*. 2014; Thapa *et al*. 2018; Lee *et al*. 2020).

## Conclusion

Each 5 cloned cells from UC with and without BRAF gene mutation (V595E) were established and examined V595E-related tumorigenic characteristics in dogs. No typical morphological features were observed in cloned cells with and without V595E. Both cell proliferation rate (doubling time) and tumorigenicity (xennotransplanted tumor growth) in cloned cells with V595E ( +) were similar to those with V595E ( −). The xenotransplanted tumors with V595E ( +) revealed typical features of UC and expressed nuclear pERK1/2, which probably indicate advanced stage of tumor growth. Coincidence of V595E with pBRAF is an important factor for acquired resistance to BRAF inhibitors. These established UC cell lines, especially V595E ( +) cell lines, are useful tool for understanding of pathophysiological states and controlling of therapeutic manners of UC in dogs.
